# ADA2 Deficiency Mimicking Acute Disseminated Encephalomyelitis

**DOI:** 10.1007/s10875-022-01413-3

**Published:** 2022-12-06

**Authors:** Lisa Ehlers, Giorgia Bucciol, KU Leuven - UZA DADA2 team, Leen Moens, Anneleen Hombrouck, Selket Delafontaine, Benson Ogunjimi, Matthias De Wachter, Diane Beysen, Isabelle Meyts

**Affiliations:** 1 Department of Immunology and Microbiology, Inborn Errors of Immunity, KU Leuven, Louvain, Belgium; 2 Childhood Immunology, Department of Pediatrics, University Hospitals Leuven, Herestraat 49, 3000 Louvain, Belgium; 3 Department of Pediatrics, Antwerp University Hospital, Antwerp, Belgium

 To the Editors,

Acute disseminated encephalomyelitis (ADEM) is an inflammatory, demyelinating condition usually affecting children and young adults days to weeks after an acute infection or vaccination [1]. Radiologically, multifocal or diffuse gray and white matter damage and increased intensity lesions on T2/FLAIR magnetic resonance imaging (MRI) sequences are found [1, 2]. ADEM typically manifests with encephalopathy, multifocal neurological deficits often affecting the cranial nerves, and headache. Less common presentations include stroke-like episodes and developmental delay [2]. ADA2 deficiency (DADA2) represents an important differential diagnosis in pediatric patients manifesting stroke-like features [3]. DADA2 is a hereditary autoinflammatory disease caused by biallelic deleterious/loss of function mutations in the *ADA2* gene [4]. Neurological manifestations are present in 51% of DADA2 patients [5].

Here, we report the case of a patient homozygous for a novel splice site variant in the *ADA2* gene who initially presented with suspected ADEM.

The patient was born to consanguineous parents (first cousins) of Turkish descent. At 7 months, he was hospitalized with encephalopathy following a febrile upper respiratory tract infection. Blood tests were unremarkable. Cerebrospinal fluid (CSF) analysis revealed pleocytosis (53 leucocytes/µL) with mononuclear cell predominance and normal glucose, lactate, and proteins. CSF cultures and PCRs for herpes simplex (HSV) and enterovirus were negative. A brain CT scan and an electroencephalogram were normal. Over the course of 4 days, he developed left-sided hemiparesis. Brain MRI revealed bilateral T2-hyperintense edema in the caudate and lentiform nuclei (Fig. 1A). A repeat lumbar puncture showed 30 mononuclear cells/µL, normal glucose, lactate, proteins, and IgG index. CSF culture and PCRs for HSV, CMV, EBV, Borrelia, and varicella-zoster (VZV) were negative. The patient was diagnosed with post-infectious ADEM and treated with high-dose intravenous glucocorticoids with slow improvement of his symptoms. Three months later, he had no focal neurological abnormalities although a transient developmental delay was noted in the recovery period. A scheduled follow-up MRI appointment was not kept.

**Fig. 1:**
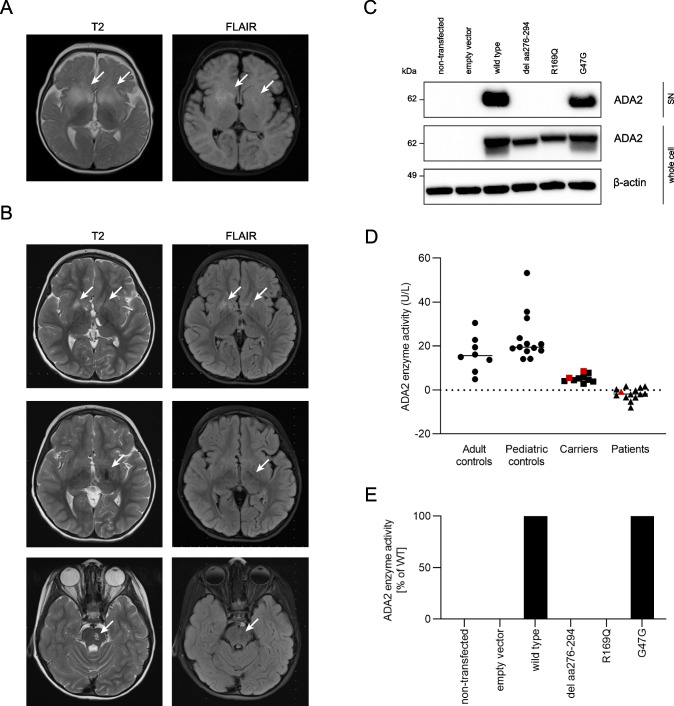
(**A**) FLAIR and T2 weighted brain MRI images at the age of 7 months showing bilateral T2-hyperintense edema in the caudate and lentiform nuclei. (**B**) FLAIR and T2 weighted brain MRI images at the age of 4.5 years, showing bilateral gliosis and tissue loss in the lentiform and caudate nucleus (upper part), the left thalamus (middle part), and the left pons (lower part), suggestive of cerebrovascular accidents. (**C**) Western blot of ADA2 in whole cell lysates and supernatants of HEK293T cells transfected with the different ADA2 variants indicated. Cells and supernatants were collected 48 h after transfection. (**D**) ADA2 enzyme activity was measured in serum samples from adult healthy controls (*n* = 8), pediatric healthy controls (*n* = 13), heterozygous carriers of ADA2 mutations (*n* = 10), and DADA2 patients (*n* = 14). The described patient and his parents are represented by the red symbols. (**E**) ADA2 enzyme activity was measured in the supernatant of HEK293T cells overexpressing the different ADA2 variants indicated. Enzyme activity is depicted relative to supernatant of HEK293T cells transfected with wild-type *ADA2* plasmid. SN, supernatant

At 3.5 years of age, he developed left ocular paresis and gait disturbances. A brain MRI at first only showed residual gliosis in the region that showed edema at 7 months of age. However, a new MRI scan performed 1 week later because of increasing somnolence revealed new ischemic lesions involving the left pons and thalamus. Treatment with intravenous glucocorticoids for suspected ADEM was initiated, which led to a rapid improvement of the gait disturbances. During this period, the patient also started experiencing recurrent fever up to 40 °C. At 4.5 years of age, the patient was admitted to the hospital for frequent postprandial vomiting, worsening gait disturbances, and recurrent pain in the hands and feet. A livedoid rash of the arms and legs was observed. An elevated CRP of 38 mg/L was noted. Brain MRI showed bilateral gliosis and tissue loss in the area of the lentiform and caudate nucleus (Fig. 1B–upper part). Additionally, areas of tissue loss were seen in the left thalamic (Fig. 1B–middle part) and pontine region (Fig. 1B–lower part), suggestive of previous cerebrovascular accidents, possibly in the context of vasculopathy rather than ADEM. At this point, the patient was referred with the suspicion of ADA2 deficiency. Serum ADA2 enzyme activity was decreased, and *ADA2* gene sequencing revealed a homozygous mutation, confirming the diagnosis of DADA2. Upon diagnosis, the patient was started on a tumor necrosis factor-α inhibitor (TNFi). This treatment was however terminated by the parents. At the time of diagnosis, the expression of interferon-stimulated genes (ISGs) in whole blood samples from the patient was strongly elevated (Supplementary Figure S4). At this point, his CRP was moderately elevated at 17 mg/L. The interferon signature decreased over time despite termination of TNFi therapy. Clinically, the patient has been stable without specific immunomodulatory treatment, at least until the time of writing.

## Identification and Characterization of a Novel ADA2 Splice Variant

Sanger sequencing revealed a novel c.881 + 1G > A (g.17672572C > T) homozygous splice site mutation in *ADA2* (NM_001282225.1), inherited from the heterozygous parents (Supplementary Figures S1-S2). The mutation locates to intron 5 of the *ADA2* gene and is predicted to cause a loss of a constitutive donor splice site according to the alternative splice site predictor (E1). Sequencing of patient mRNA-derived cDNA amplified by qPCR revealed an inframe deletion of amino acids 276–294 (c.825–881, exon 5) in the catalytic domain of ADA2 (Supplementary Table S1). Accordingly, overexpression of the variant in HEK293T/17 cells led to a 2 kDa smaller protein in comparison with wildtype ADA2 as verified by Western blot (Fig. 1C). In contrast with wildtype protein, the splice variant cannot be detected in the supernatant of transfected HEK293T/17 cells (Fig. 1C), suggesting impaired secretion of the mutant protein. Additionally, gel electrophoresis of ADA2-specific PCR products amplified from patient cDNA did not yield the expected band hinting at nonsense-mediated *ADA2* mRNA decay in the patient (Supplementary Figure S3).

The serum ADA2 enzyme activity of the patient was reduced to the level found in patients with confirmed ADA2 deficiency (Fig. 1D). Measurements from the parents’ serum were in line with their heterozygous carrier status (Fig. 1D). ADA2 enzyme activity was not measurable in supernatant from HEK293T/17 cells transfected with the c.881 + 1 G > A variant while enzymatically active ADA2 was found after overexpression of wildtype and the sham variant G47G (Fig. 1E). In conclusion, the in vitro analyses confirmed the pathogenicity of the identified novel variant in the *ADA2* gene. Materials and methods for the presented findings are provided in the supplementary material.

In this paper, we describe a patient presenting with encephalopathy and multifocal neurological deficits mistaken for ADEM who was diagnosed with ADA2 deficiency due to a novel splice variant in the *ADA2* gene (E2). Typical manifestations of ADEM include acute hemiparesis, pyramidal signs, slurred speech, aphasia, and ataxia, largely overlapping with those of ischemic or hemorrhagic strokes in DADA2 [4] (E3). Similarly, lymphocytic pleocytosis has been observed in the CSF of both patients with ADEM and DADA2 [4] (E4). Especially in patients with additional symptoms hinting at a systemic disease including fever, livedoid rash, and elevated acute phase reactants, the screening threshold for DADA2 should be low. In our patient, the suspicion of DADA2 only arose after a second episode of suspected ADEM with the development of recurrent fever episodes and a livedoid rash. However, in retrospect, several features of his presentation were not characteristic of typical ADEM and, taken together with his parents’ consanguinity, would have warranted an earlier genetic evaluation. At 7 months of age, his initial manifestation was decidedly below the average age of ADEM onset at 5 to 8 years [2]. Additionally, the predominant gray matter affection and the multiphasic character of his disease have to be noted as atypical [1].

A timely diagnosis is crucial to prevent potential long-term sequelae due to recurrent strokes. Early initiation of immunomodulatory treatment with TNFi has been shown to prevent ischemic events in DADA2 patients (E5). By and large experts are in agreement that upon the diagnosis of DADA2, also in the absence of central nervous system manifestations, TNFi should be initiated, even in early childhood, if possible. This is especially true as the onset of disease is usually in childhood with around 25% of patients presenting prior to 1 year of age and with ischemic stroke described in patients only a few months old [5]. If the necessary precautions are taken, TNFi are mostly well tolerated, at least in DADA2. In our patient, the parents stopped the treatment shortly after the diagnosis. Fortunately, the boy has been clinically well. Also, we saw a spontaneous reduction in his interferon signature over time. Elevation of interferon-stimulated genes is a well-known feature of the immunological phenotype of DADA2 (E6). Although long-term data are sparse, a correlation between interferon score and disease severity as well as acute phase reactants has been reported (E7). The same article described a reduction in interferon score upon initiation of TNFi treatment, while it remained elevated in the cohort described by Nihira et al. (E7, E8). Further research is needed to determine whether the type I interferon signature in DADA2 correlates with disease activity.

In conclusion, we here describe a patient who was diagnosed with ADA2 deficiency following initial presentation with ADEM-like disease. In this patient, we identified a novel pathogenic splice variant in the *ADA2* gene.

## Supplementary Information

Below is the link to the electronic supplementary material.

Supplementary file1 (DOCX 1026 KB)

## Data Availability

All data generated or analysed during this study are included in this published article and its supplementary information files.
