# Supplementary material for: ADA2 Deficiency Mimicking Acute Disseminated Encephalomyelitis
Source: J Clin Immunol. 2022 Dec 6;43(3):536–9. doi: 10.1007/s10875-022-01413-3 (PMC9957855; doi:10.1007/s10875-022-01413-3)
Supplement: Supplementary file 1 — Supplementary file1 (DOCX 1026 KB) [file 10875_2022_1413_MOESM1_ESM.docx]

Supplementary material

[Methods 1](#_Toc112837638)

[DNA sequencing 1](#_Toc112837639)

[Gene expression 2](#_Toc112837640)

[Interferon-stimulated gene signature 2](#_Toc112837641)

[In vitro analysis of ADA2 variants 2](#_Toc112837642)

[ADA2 enzyme activity 3](#_Toc112837643)

[Ethical approval 3](#_Toc112837644)

[Supplementary Figures 3](#_Toc112837645)

[Supplementary Figure S1 3](#_Toc112837646)

[Supplementary Figure S2 4](#_Toc112837647)

[Supplementary Figure S3 5](#_Toc112837648)

[Supplementary Figure S4 5](#_Toc112837649)

[Supplementary Tables 6](#_Toc112837650)

[Supplementary Table S1: pJET1.2 cloning of ADA2-specific PCR products from patient cDNA 6](#_Toc112837651)

Supplementary [references 8](#_Toc112837652)

# Methods

## DNA sequencing

Genomic DNA samples were prepared from heparinised peripheral blood using the QIAamp DNA Blood Mini kit (QIAGEN, Hilden, Germany). Oligo Primer analysis software version 7 (Molecular Biology Insights, Inc., Casade, CO, USA) was used for primer design. ADA2-specific gDNA amplification was performed using Platinum SuperFi PCR Master mix (Thermo Fisher Scientific, Merelbeke, Belgium). PCR products were purified using QIAquick PCR purification kit (Qiagen, Hilden, Germany). Sanger sequencing was performed on an ABI 3730 XL Genetic Analyzer (Applied Biosystems, Foster City, Calif) at LGC Genomics (Berlin, Germany). Sequencing data were analysed using Chromas 2.6.5 (http://www.technelysium.com.au). No DNA samples were available from healthy siblings.

## Gene expression

Total RNA was extracted from PAXgene RNA tubes using the PAXgene Blood RNA Kit, v2 (PreAnalytiX, Qiagen/ BD). mRNA was reverse-transcribed directly with the Superscript Vilo cDNA synthesis kit (Thermo Fisher Scientific). To evaluate the potential impact of the investigated variant, an ADA2 cDNA-specific PCR fragment spanning exons four to seven was produced with the following primers: ADA2_EX4_F GGTCTCATCCATTACGCACCA and ADA2_EX7_R GTAAGGCAGCTTAACGCCAT. PCR-purified products were cloned using the CloneJET PCR cloning kit (Thermo Fisher Scientific). After transformation, pJET1.2 plasmids were isolated with the QIAprep Spin miniprep kit (Qiagen, Hilden, Germany) and sequenced at LGC Genomics (Berlin, Germany).

## Interferon-stimulated gene signature

RNA was extracted from whole blood as described above. cDNA was generated 40 ng RNA using the Superscript Vilo cDNA synthesis kit (Thermo Fisher Scientific). Quantitative polymerase chain reaction (qPCR) analysis was performed using SsoAdvance Universal Sybr Green Supermix (Bio-Rad Laboratories, Inc., Hercules, California) and the following primers: IFI27_F_TCGCCTCGTCCTCCATAGCAG; IFI27_R_AGTAGAACCTCGCAATGACAGCC; IFI44L_F_ATCTTAAAAGGTTGTATGCCAGA; IFI44L_R_ACTTGCTTCACTTTTGCCAA; IFIT1_F_ATGAGTACAAATGGTGATGA; IFIT1_R_AATTCAATCTGATCCAAGAC; ISG15_F_GGTGGACAAATGCGACGAACCTC; ISG15_R_CACACCCTCCAGCCCGCTCA; RSAD2_F_GCGTCAACTATCACTTCACTCG; RSAD2_R_CAGGTATTCTCCCCGGTCT; SIGLEC1_F_TCTTGCCCAAGCTTCTCCTC; SIGLEC1_R_GTAGTACCAGATGGCCGTGA; GAPDH_F_GTCTCCTCTGACTTCAACAGCG; GAPDH_R_ACCACCCTGTTGCTGTAGCCAA. All conditions were measured in three technical replicates. The reaction was run on a QuantStudio™ 3 Real-Time PCR System (Thermo Fisher Scientific) and analysed using the QuantStudio™ Design & Analysis Software v1.5.2. The relative abundance of each transcript was normalised to the expression level of GAPDH. The median fold change of the six interferon-stimulated genes (ISGs) when compared to expression in healthy control samples was used to create an interferon score (IS) for each patient.(1) IS is equal to 2^-ΔΔCt^ i.e. the normalized fold change relative to healthy control.

## In vitro analysis of ADA2 variants

The pCMV6-c-myc plasmid expressing wildtype ADA2 (NM_001282225, #RC238645) was obtained from OriGene Technologies Inc. (Rockville, Maryland). Site-directed mutagenesis was performed using the NEB Q5 mutagenesis kit (New England Biolabs, Ipswich, Massachusetts). Stable competent E. coli (#C3040H, New England Biolabs) were transformed with the constructs and plasmid DNA was purified using QIAprep Spin Miniprep Kit (QIAGEN, Hilden, Germany). Successful mutagenesis was verified by Sanger sequencing (LGC Genomics, Germany). Plasmids were transfected into HEK293T/17 cells (ATCC, Manassas, Virginia), using X-tremeGENE 9DNA transfection reagent (Merck KGaA, Germany). Cells and supernatant were collected 72 hours after transfection. Transfection efficiency was evaluated by qPCR (see above for the detailed protocol) using the following primers: ADA2_F_ACCAGAATCGGCCATGGA; ADA2_R_CTACAGGGTGGTTCCTCAAGTCA. Transfected HEK293T/17 cells were lysed with RIPA buffer. Protein expression in whole cell extracts and supernatant was evaluated on western blot using the following antibodies: anti-ADA2 (EPR25430-131, Abcam, Cambridge, United Kingdom), anti-beta-actin (AC-15, Sigma-Aldrich, St. Louis, Missouri; RRID: AB_476744), anti-DDK (OTI4C5, OriGene; RRID: AB_2622345), goat anti-rabbit-HRP (Abcam; RRID: AB_2819160), goat anti-mouse-HRP (Merck Millipore, Burlington, Massachusetts; RRID: AB_11211441). Visualisation was achieved by enzymatic chemiluminescence using ECL western blotting substrate (Thermo Fisher Scientific) in a ChemiDoc XRS+ Imaging System (Bio-Rad).

## ADA2 enzyme activity

Adenosine deaminase 2 activity in serum and supernatant from transfected HEK293T/17 was measured in a colorimetric assay adapted from Giusti and Galanti.(2) Erythro-9-(2-hydroxy-3-nonyl) adenine (EHNA) (Sigma-Aldrich) was used as an inhibitor of ADA1 activity. Each sample was analysed in triplicate. Measurements for ADA2 variants in supernatant from transfected HEK293T/17 cells were normalised to the activity of wildtype ADA2.

## Ethical approval

The Ethics Committee of the University Hospital Leuven approved this study (protocol number: S63077). The study was performed in compliance with the Declaration of Helsinki for research involving human subjects. Written informed consent was obtained from the parents prior to DNA/RNA isolation and serum preparation from blood of the tested family members and healthy controls.

## Methods references

1. Rice GI, Melki I, Frémond ML, Briggs TA, Rodero MP, Kitabayashi N, et al. Assessment of Type I Interferon Signaling in Pediatric Inflammatory Disease. J Clin Immunol. 2017 Feb 1;37(2):123–32.

2. Illingworth J. Methods of enzymatic analysis: Third edition: Editor-in-Chief: Hans Ulrich Bergmeyer. Verlag Chemie, 1983 (vols I–III), 1984 (vols IV & V) DM258 each volume or DM2240 vols I–X inclusive. Biochemical Education. 1985;13(1):38–38.

# Supplementary Figures

## Supplementary Figure S1


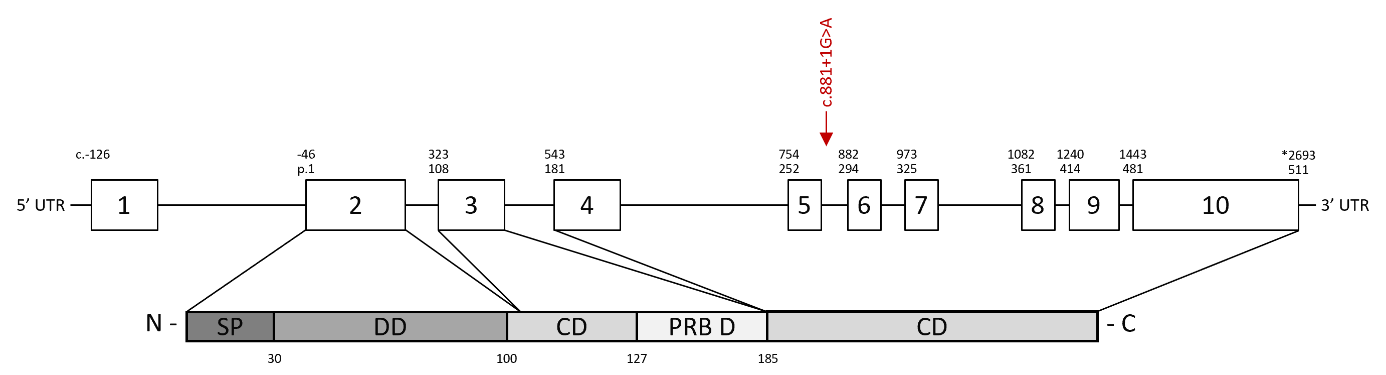


**Supplementary Figure S1:** Schematic overview of the *ADA2* gene and ADA2 protein illustrating the novel mutation in intron 5. Legend: Legend: CD, catalytic domain; DD, dimerisation domain; PRB D, putative receptor binding domain; SP, signal peptide.

## Supplementary Figure S2

**Supplementary Figure S2:** Sanger sequencing of the *ADA2* gene identified the novel homozygous splice site mutation c.881+1G>A in the patient. The parents were confirmed as heterozygous carriers of the mutation.


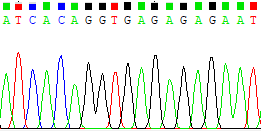

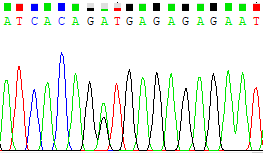

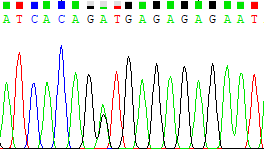

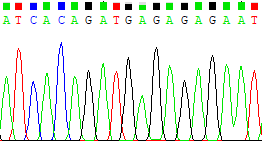


Control

Father

Mother

Patient

##

## Supplementary Figure S3


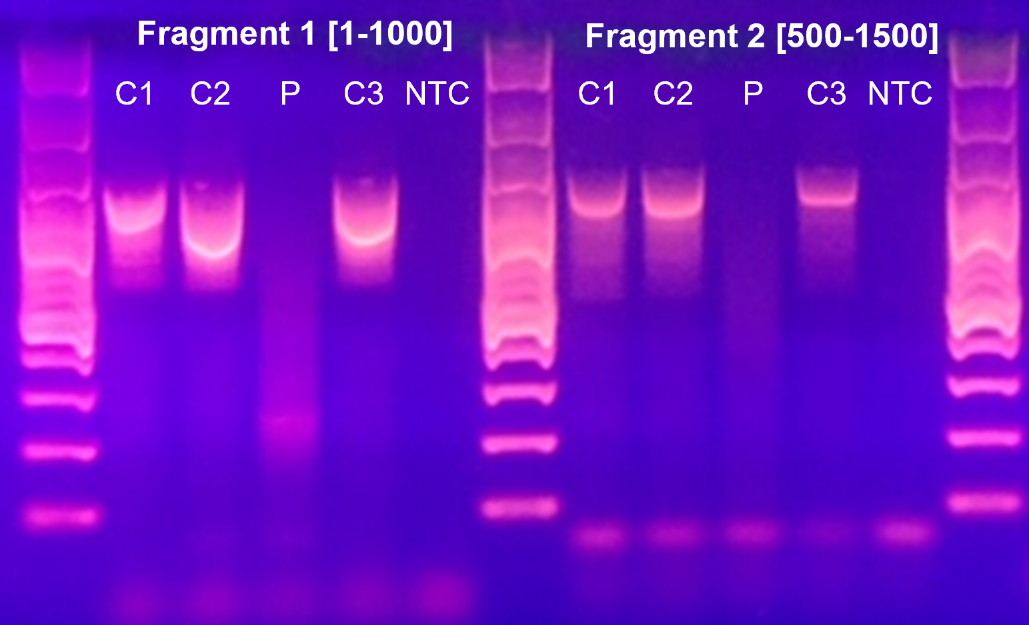


**Supplementary Figure S3:** Nonsense-mediated mRNA decay in the DADA2 patient. Agarose gel of PCR products using different *ADA2*-specific primers. cDNA was synthetised from RNA extracted from whole blood (PAXgene tubes). Legend: C, control; P, patient; NTC, no template control.

## Supplementary Figure S4

**Supplementary Figure S4:** The interferon score was determined by quantitative PCR of a panel of six interferon-stimulated genes (*IFI27*, *IFI44L*, *IFIT1*, *ISG15*, *RSAD2*, *SIGLEC1*) in whole blood samples from the patient at different time points. Gene expression was normalised to *GAPDH*. The interferon score is equal to 2^-ΔΔCt^, i.e. the normalised fold change relative to a calibrator. Legend: TNFi, TNF-α inhibitor.

# Supplementary Tables

## Supplementary Table S1: pJET1.2 cloning of ADA2-specific PCR products from patient cDNA

| pJET clones | % |
| --- | --- |
| WT | 1.6 |
| Inframe deletion of AA 276-294 | **38.1** |
| Inframe deletion of AA 261-332 | 9.5 |
| S295X | 4.8 |
| Inframe deletion of AA 228-337 | 3.2 |
| Inframe deletion of AA 232-333 | 3.2 |
| Inframe deletion of AA 236-331 | 1.6 |
| Inframe delition of AA 286-312 | 1.6 |
| Inframe deletion of AA 276-315 | 1.6 |
| Inframe deletion of AA 252-331 | 1.6 |
| Inframe deletion of AA 289-295 | 1.6 |
| Inframe deletion of AA 226-338 | 1.6 |
| Inframe deletion of AA 264-294 | 1.6 |
| Inframe deletion of AA 233-334 | 1.6 |
| Inframe deletion of AA 287-295 | 1.6 |
| Inframe deletion of AA 232-334 | 1.6 |
| Inframe deletion of AA 237-337 | 1.6 |
| Inframe deletion of AA 238-329 | 1.6 |
| Inframe deletion of AA 243-337 | 1.6 |
| D226Afs3X | 1.6 |
| Q233Tfs6x | 1.6 |
| M232Sfs9X | 1.6 |
| Y242Cfs10X | 1.6 |
| L241Pfs1X | 1.6 |
| R230Lfs2X | 1.6 |
| H260Lfs2X | 1.6 |
| M232Lfs14X | 1.6 |
| F276Wfs16X | 1.6 |
| E262Lfs3X | 1.6 |
| S256Lfs2X | 1.6 |
| N239Ifs3X | 1.6 |

# Supplemental references

E1. Wang M, Marín A. Characterization and prediction of alternative splice sites. Gene. 2006 Feb 1;366(2):219–27.

E2. Krupp LB, Banwell B, Tenembaum S. Consensus definitions proposed for pediatric multiple sclerosis and related disorders. Neurology. 2007 Apr 17;68(16 suppl 2):S7–12.

E3. Matricardi S, Farello G, Savasta S, Verrotti A. Understanding Childhood Neuroimmune Diseases of the Central Nervous System. Frontiers in Pediatrics [Internet]. 2019 [cited 2022 May 26];7. Available from: https://www.frontiersin.org/article/10.3389/fped.2019.00511

E4. Schwarz S, Knauth M, Mohr A, Wildemann B, Sommer C, Storch-Hagenlocher B. Akute disseminierte Enzephalomyelitis (ADEM). Nervenarzt. 2001 Mar 1;72(4):241–54.

E5. Cooray S, Omyinmi E, Hong Y, Papadopoulou C, Harper L, Al-Abadi E, et al. Anti-tumour necrosis factor treatment for the prevention of ischaemic events in patients with deficiency of adenosine deaminase 2 (DADA2). Rheumatology (Oxford). 2021 Sep 1;60(9):4373–8.

E6. Belot A, Wassmer E, Twilt M, Lega JC, Zeef LA, Oojageer A, et al. Mutations in CECR1 associated with a neutrophil signature in peripheral blood. Pediatr Rheumatol Online J. 2014;12:44.

E7. Insalaco A, Moneta GM, Pardeo M, Caiello I, Messia V, Bracaglia C, et al. Variable Clinical Phenotypes and Relation of Interferon Signature with Disease Activity in ADA2 Deficiency. J Rheumatol. 2019 May;46(5):523–6.

E8. Nihira H, Izawa K, Ito M, Umebayashi H, Okano T, Kajikawa S, et al. Detailed analysis of Japanese patients with adenosine deaminase 2 deficiency reveals characteristic elevation of type II interferon signature and STAT1 hyperactivation. J Allergy Clin Immunol. 2021 Jan 30;
